# Preoperative 18F-FDG PET/CT tumor markers outperform MRI-based markers for the prediction of lymph node metastases in primary endometrial cancer

**DOI:** 10.1007/s00330-019-06622-w

**Published:** 2020-02-07

**Authors:** Kristine E. Fasmer, Ankush Gulati, Julie A. Dybvik, Sigmund Ytre-Hauge, Øyvind Salvesen, Jone Trovik, Camilla Krakstad, Ingfrid S. Haldorsen

**Affiliations:** 1 Mohn Medical Imaging and Visualization Centre (MMIV), Department of Radiology, Haukeland University Hospital, Jonas Liesvei 65, 5021 Bergen, Norway; 2 Section for Radiology, Department of Clinical Medicine, University of Bergen, Bergen, Norway; 3 Unit for Applied Clinical Research, Department of Public Health and Nursing, Norwegian University of Science and Technology, Trondheim, Norway; 4 Department of Obstetrics and Gynaecology, Haukeland University Hospital, Bergen, Norway; 5 Centre for Cancer Biomarkers, Department of Clinical Science, University of Bergen, Bergen, Norway

**Keywords:** Endometrial cancer, PET-CT, 18F-FDG, Magnetic resonance imaging, Lymphatic metastasis

## Abstract

**Objectives:**

To compare the diagnostic accuracy of preoperative 18F-FDG PET/CT and MRI tumor markers for prediction of lymph node metastases (LNM) and aggressive disease in endometrial cancer (EC).

**Methods:**

Preoperative whole-body 18F-FDG PET/CT and pelvic MRI were performed in 215 consecutive patients with histologically confirmed EC. PET/CT-based tumor standardized uptake value (SUV_max_ and SUV_mean_), metabolic tumor volume (MTV), and PET-positive lymph nodes (LNs) (SUV_max_ > 2.5) were analyzed together with the MRI-based tumor volume (V_MRI_), mean apparent diffusion coefficient (ADC_mean_), and MRI-positive LN (maximum short-axis diameter ≥ 10 mm). Imaging parameters were explored in relation to surgicopathological stage and tumor grade. Receiver operating characteristic (ROC) curves were generated yielding optimal cutoff values for imaging parameters, and regression analyses were used to assess their diagnostic performance for prediction of LNM and progression-free survival.

**Results:**

For prediction of LNM, MTV yielded the largest area under the ROC curve (AUC) (AUC = 0.80), whereas V_MRI_ had lower AUC (AUC = 0.72) (*p* = 0.03). Furthermore, MTV > 27 ml yielded significantly higher specificity (74%, *p* < 0.001) and accuracy (75%, *p* < 0.001) and also higher odds ratio (12.2) for predicting LNM, compared with V_MRI_ > 10 ml (58%, 62%, and 9.7, respectively). MTV > 27 ml also tended to yield higher sensitivity than PET-positive LN (81% vs 50%, *p* = 0.13). Both V_MRI_ > 10 ml and MTV > 27 ml were significantly associated with reduced progression-free survival.

**Conclusions:**

Tumor markers from 18F-FDG PET/CT outperform MRI markers for the prediction of LNM. MTV > 27 ml yields a high diagnostic performance for predicting aggressive disease and represents a promising supplement to conventional PET/CT reading in EC.

**Key Points:**

*• Metabolic tumor volume (MTV) outperforms other 18F-FDG PET/CT and MRI markers for preoperative prediction of lymph node metastases (LNM) in endometrial cancer patients.*

*• Using cutoff values for tumor volume for prediction of LNM, MTV > 27 ml yielded higher specificity and accuracy than V*_*MRI*_*> 10 ml.*

*• MTV represents a promising supplement to conventional PET/CT reading for predicting aggressive disease in EC.*

**Electronic supplementary material:**

The online version of this article (10.1007/s00330-019-06622-w) contains supplementary material, which is available to authorized users.

## Introduction

Endometrial cancer is the sixth most common cancer among women worldwide, and the incidence has been steadily increasing over the past decades [1]. Endometrial tumors are histologically classified as non-endometrioid subtype or endometrioid subtype (grades 1–3), and non-endometrioid subtype and grade 3 endometrioid subtype are associated with high-risk disease [2]. Endometrial cancer is surgicopathologically staged according to The International Federation of Gynecology and Obstetrics (FIGO) system, with evaluation of tumor extent and lymph node involvement [3]. Presence of lymph node metastases (LNM) implies poorer prognosis, and the preoperative identification of patients at high risk of having LNM may be useful for tailoring lymphadenectomy and subsequent adjuvant therapy. Routine lymphadenectomy is controversial due to lack of evidence supporting that this improves survival [4–6], paralleled by well-known side effects from lymphadenectomy with resulting reduced quality of life. Preventing surgical over- and under-treatment by tailoring lymphadenectomy only to patients at high risk of extrauterine disease, is thus crucial if improved endometrial cancer patient care is to be achieved.

Different prediction models for LNM in endometrial cancer have been suggested. Some models are inherently postoperative since they are based on tumor biomarker profiles derived from hysterectomy specimens [7–9], whereas proposed preoperative models combine preoperative imaging characteristics and biopsy/curettage and serum markers, e.g., cancer antigen (CA 125) [10–12]. When applied in independent patient cohorts, these models have been shown to have variable feasibilities [13–15], and at present, the best risk stratification model in endometrial cancer is not yet defined, and no uniform risk model is routinely used across centers. Furthermore, sentinel lymph node dissection (SLND) procedures have been increasingly advocated as a feasible alternative to full lymphadenectomy in endometrial cancer patients. However, how to select patient groups that are likely to benefit from SLND and how the procedure is optimally performed are not yet fully known [2, 16, 17].

Contrast-enhanced (CE) magnetic resonance imaging (MRI) has long been considered the radiological imaging method of choice for preoperative assessment of local tumor stage in endometrial cancer (i.e., the identification of deep myometrial invasion, cervical stroma invasion, and pelvic LNM). However, the reported diagnostic staging performance of CE MRI has a broad range and well-known limitations in particular for diagnosing LNM [18]. Preoperative 18F fluorodeoxyglucose (18F-FDG) positron emission tomography combined with computed tomography (PET/CT) is increasingly used for staging of various cancers, including endometrial cancer. Several studies on small- to medium-sized cohorts have reported a high diagnostic performance of 18F-FDG PET/CT in endometrial cancer, especially for detecting LNM [19–23].

The primary objectives of this study were to assess and compare the diagnostic accuracy of preoperative 18F-FDG PET/CT- and MRI-derived markers for the prediction of LNM and aggressive disease in a large endometrial cancer patient cohort. Furthermore, this study aimed to explore how metabolic tumor parameters from 18F-FDG PET/CT- and MRI-based tumor markers are interrelated.

## Materials and methods

### Patient series and study setting

This retrospective cohort study was conducted under institutional review board (IRB)–approved protocols with written informed consent from all patients. Preoperative pelvic MRI and whole-body 18F-FDG PET/CT were performed from October 2011 to December 2016 in all patients, *n* = 215, with histologically confirmed endometrial carcinoma at surgery. All patients had a single primary tumor. Mean (range) time span between MRI and 18F-FDG PET/CT was 4 (0–38) days. The mean (range) time interval between MRI examination and primary treatment was 16 (0–98) days and between PET/CT and primary treatment 16 (0–102) days. The shortest interval (zero days) between preoperative imaging and treatment was recorded in two patients having inoperable disease (FIGO IV) who started chemotherapy at the day of the imaging examination. All patients were diagnosed and treated at the same university hospital serving a population of ~ 1 million inhabitants. Clinical data (e.g., age, menopausal status, height, body weight) were registered, and patients were staged according to the FIGO 2009 criteria [3]. Depths of myometrial invasion (MI), cervical stroma invasion (CI), and lymph node metastases (LNM) were evaluated by the pathologists using standard procedures. Patient follow-up data have been collected from patient records and from correspondence with the responsible physicians/gynecologists. For patients considered radically treated (203/215 patients), standard-of-care follow-up is clinical examinations quarterly during the first 2 years and biannually until 5 years after primary diagnosis. For patients not considered radically treated (12/215 patients), the follow-up is individualized, normally with frequent follow-ups. Mean (range) follow-up time for survivors was 33 (0–66) months and date of last follow-up was 16 August 2018. Progression was defined as local recurrence/progression in the pelvis or new metastases in the abdomen or at distant locations.

### MRI protocol and image analysis

Pelvic MRI was acquired on a Siemens Avanto 1.5-T scanner for 156/215 patient and on a Siemens Skyra 3-T scanner for the remaining 59/215 patients. Prior to imaging, 20 mg butylscopolamine bromide (Buscopan, Boehringer Ingelheim) was administered intravenously to reduce bowel peristalsis. The MRI protocol included sagittal and axial oblique (perpendicular to the long axis of the uterus) T2-weighted images and axial oblique T1-weighted gradient-echo images before and 2 min after administration of 0.1 mmol gadolinium/kg body weight (Dotarem, Guerbet). Diffusion-weighted imaging (DWI) was performed using an axial oblique 2D echo-planar imaging sequence with *b* values of 0 and 1000 s/mm^2^, and apparent diffusion coefficient (ADC) maps were generated. Information on 1.5-T and 3-T scanner protocols are given in Suppl. Table 1.

**Fig. 1:**
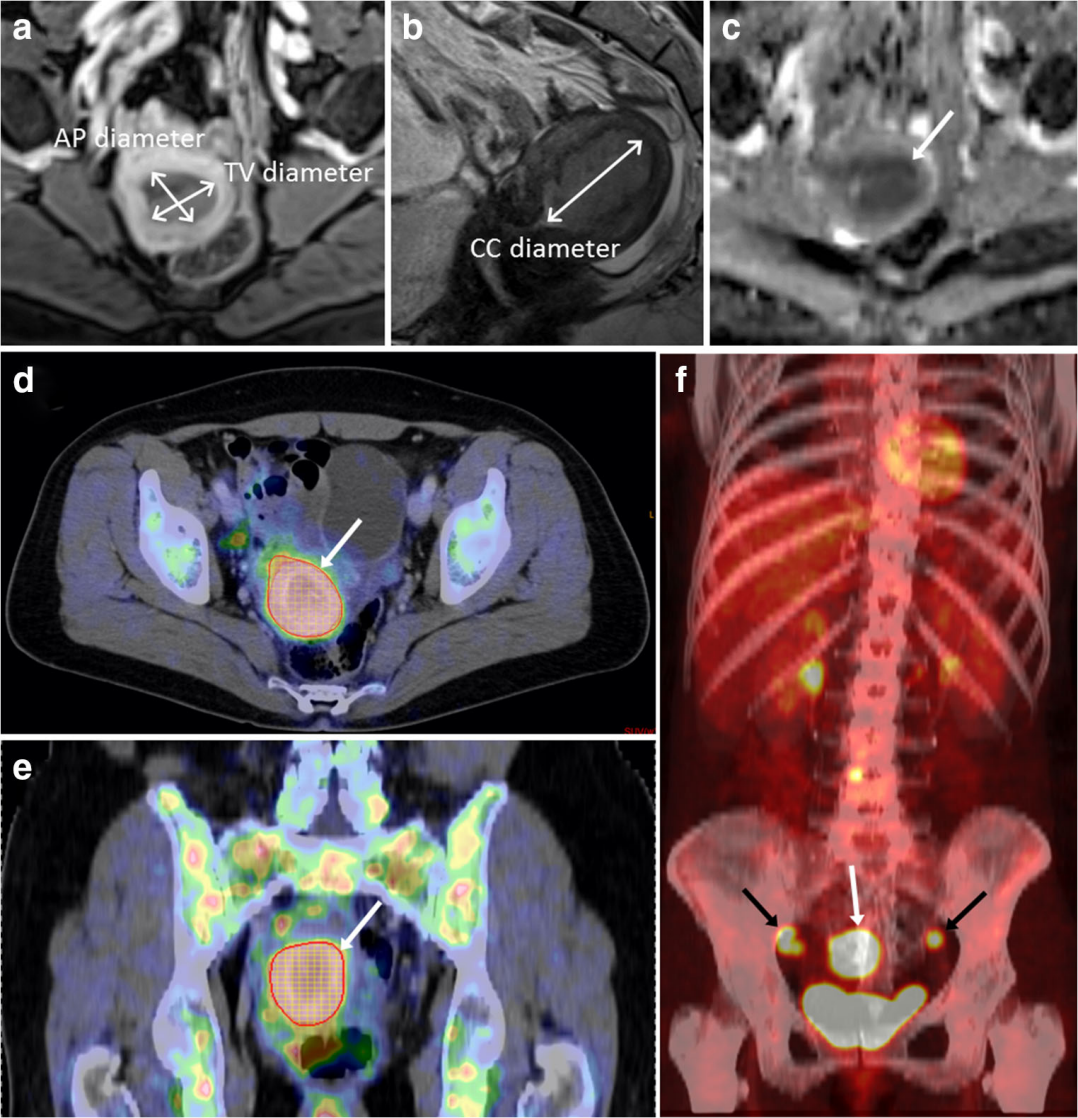
Pelvic MRI (**a**–**c**) and whole-body 18F-FDG PET/CT (**d**–**f**) of a 60-year-old patient with endometrial carcinoma, FIGO stage IIIC (endometrioid, grade 3). **a** Axial oblique contrast-enhanced T1-weighted MRI and (**b**) sagittal T2-weighted MRI for measurement of maximum tumor diameters in three orthogonal planes (AP, TV, and CC diameters). **c** On apparent diffusion coefficient map, the tumor (arrow) is depicted as hypointense indicating restricted diffusion. **d** Axial and (**e**) frontal 18F-FDG PET/CT show the metabolic tumor volume (MTV) (red line shows the MTV boundaries) in which the maximum and mean standardized uptake values are measured. **f** Maximum intensity projection map depicts the 18F-FDG-avid primary tumor (white arrow) and 18F-FDG-avid pelvic metastatic lymph nodes (black arrows)

The de-identified MRI images were evaluated by three radiologists with 2–10 years of experience, who were blinded to clinical data, tumor stage, and patient outcome. Maximum tumor diameter was measured in three orthogonal planes: anteroposterior (AP) and transverse (TV) diameters on CE paraxial T1-weighted images and the craniocaudal (CC) tumor diameter on sagittal T2-weighted images (Fig. 1). Tumor volume (V_MRI_) was estimated by assuming the shape of an ellipsoid: V_MRI_ = 4/3*π*(*AP*/2 × *TV*/2 × *CC*/2). In addition, for a subgroup of 60 patients (randomly selected), 3D tumor masks were outlined by one of the radiologists (JAD) in order to compare the MRI tumor volume estimated by the ellipsoid method with a supposedly more exact 3D tumor volume measurement. MRI findings suggesting deep (≥ 50%) myometrial invasion (DMI) and pelvic or paraaortic LNM (MRI-positive lymph nodes (LN), defined as enlarged LN with a maximum short-axis diameter of ≥ 10 mm) were recorded by all three readers. Since this study was focused on patient-based analysis, the number, shape, and position of the enlarged LNs were (although registered) not taken into account for prediction of LNM at surgical staging. The mean tumor apparent diffusion coefficient (ADC_mean_) was measured in a region of interest (ROI) drawn in the ADC map depicting the largest cross-sectional tumor diameter. Consensus values for the three readers were established using the median value for continuous variables and the majority reading for categorical variables.

### 18F-FDG PET/CT imaging protocol and image analysis

18F-FDG PET/CT was performed on a Siemens Biograph 40 True Point scanner, with scan range coverage from the skull base to the mid-thigh. All patients were instructed to fast for 6 h prior to scanning and had an i.v. injection of 4.6 MBq 18F-FDG/kg body weight or 370 MBq 18F-FDG approximately 60 min prior to scanning. The PET images were acquired with 3 min per bed position and reconstructed with correction for scatter and attenuation based on the CT images. The CT protocol was changed during the study period, from diagnostic CE CT to low-dose CT. Thus, attenuation correction of the PET signal was performed using diagnostic CE CT (120 kV, 240 reference mAs) in 11/215 patients and low-dose CT (120 kV and 50 reference mAs) in 204/215.

A physician with > 2 years of PET/CT experience and who was blinded for clinical findings, MRI findings, and surgical staging results reviewed all images retrospectively on a Segami Oasis workstation. Metabolic tumor volume (MTV) was calculated by segmenting a volume of interest (VOI) including all putative tumor voxels with body weight standardized uptake value (SUV) > 2.5. The selection of SUV threshold was based on clinical experiences of SUV levels in healthy background tissue. The mean and maximum SUV values within this VOI, SUV_mean_, and SUV_max_, respectively, were also recorded in addition to the presence of increased 18F-FDG uptake in lymph nodes (SUV_max_ > 2.5), interpreted as likely LNM (PET-positive LN) (Fig. 1).

### Statistical analysis

Median values with 95% confidence interval (CI) were assessed for all imaging-derived tumor parameters. Correlation analyses were performed using Spearman’s rank-order correlation test, and interobserver reliability was assessed using intraclass correlation coefficient (ICC) for continuous variables and Fleiss kappa statistics for categorical variables. The Mann-Whitney *U* test was used to analyze differences in imaging parameters in relation to surgicopathological stage and tumor grade. Receiver operating characteristic (ROC) analyses were employed to compare the diagnostic performance of the different imaging parameters for predicting LNM, and optimal cutoff values for MTV and V_MRI_ were identified from the ROC curves using the Youden index. Patient-based logistic regression analysis for prediction of LNM was conducted for the categorized imaging variables as well as for the detection of LNM on conventional PET/CT and MRI reading (PET- and MRI-positive LN). Sensitivity, specificity, and accuracy were compared by McNemar’s test. The prognostic value of the imaging parameters was explored using the Kaplan-Meier with log-rank test and the Cox proportional hazard model. Analyses were performed in SPSS 24.0 (IBM Corp.) and STATA 15.1 (StataCorp). The reported *p* values were generated by two-sided tests and considered significant when less than 0.05.

## Results

### Patients and treatment

A total of 215 patients with endometrial cancer were included in the study (Table 1), and all patients were treated according to the Norwegian national guidelines for endometrial cancer. Altogether, 98% (211/215) of the patients underwent primary surgical resection with hysterectomy and bilateral salpingo-oophorectomy. One patient (1/215) underwent tumor debulking without hysterectomy (grade 3, FIGO IVB); two patients (2/215) were deemed medically ineligible for surgical treatment (both grade 3, FIGO IVA and IVB, respectively); and one patient (1/215) who wished to preserve fertility, refrained from surgery (grade 1, FIGO IA). The histological diagnoses of these four patients are based on uterine biopsies and recorded FIGO stage on findings from diagnostic imaging.

**Table 1:** Patient characteristics and surgicopathological findings in 215 endometrial cancer patients

Age, mean (range)	68 (30–90)
BMI, mean (range)	29 (16–53)
Postmenopausal, *n* (%)	204 (95%)
Risk status* from preoperative biopsy/curettage	
Low risk	144 (67%)
High risk	71 (33%)
FIGO stage^†^, *n* (%)	
Stage IA	123 (57%)
Stage IB	48 (22%)
Stage II	18 (8%)
Stage IIIA	3 (1%)
Stage IIIB	4 (2%)
Stage IIIC1	12 (6%)
Stage IIIC2	1 (1%)
Stage IVA	1 (1%)
Stage IVB	5 (2%)
Histologic subtype^†^, *n* (%)	
Endometrioid	172 (80%)
Non-endometrioid	39 (18%)
Undifferentiated/other	4 (2%)
Histologic grade^†^ (endometrioid only), *n* (%)	
Grade 1	98 (57%)
Grade 2	42 (24%)
Grade 3	27 (16%)
Missing	5 (3%)
Myometrial invasion^†^, *n* (%)	
< 50%	140 (65%)
≥ 50%	72 (34%)
Missing	3 (1%)
Cervical stroma invasion^†^, *n* (%)	
No	180 (84%)
Yes	31 (14%)
Uterus not removed	4 (2%)
Lymphadenectomy^†^, *n* (%)	
Pelvic	95 (44%)
Pelvic + paraaortic	43 (20%)
No	77 (36%)
Lymph node metastases^†^, *n* (%)	
No	122 (57%)
Yes	16 (7%)
Not investigated	77 (36%)

*BMI*, body mass index; *FIGO*, International Federation of Gynecology and Obstetrics

*Low risk: endometrioid subtype grades 1 and 2; high risk: endometrioid subtype grade 3 and non-endometrioid subtype

^†^Findings from surgical and pathological staging

Pelvic lymph node dissection (PLND) was performed in 138 patients and revealed LNM in 12% (16/138) of the patients while paraaortic lymph node dissection (PALND) in 43 patients revealed paraaortic LNM in 2% (1/43). Median (range) of nodes examined was 13 (1–35) for PLND and 19 (5–54) for PALND. Patients undergoing lymphadenectomy had significantly lower BMI, and a higher proportion of the patients had high-grade tumors, non-endometrioid subtype, DMI; received adjuvant treatment; and experienced disease progression (Suppl. Table 2).

Adjuvant therapy was given in 36% (78/215) of the patients; chemotherapy in 33% (72/215), external radiation in 1% (2/215), internal radiation in 1% (2/215), and hormonal treatment in 1% (2/215).

### Interobserver reproducibility for MRI assessment and intercorrelation between MRI and PET/CT tumor parameters

The interobserver reliability for the MRI tumor measurements assessed by the three readers was high with ICC (95% CI) of 0.947 (0.934–0.958) for AP diameter, 0.912 (0.879–0.935) for TV diameter, 0.942 (0.923–0.957) for CC diameter, and 0.919 (0.897–0.937) for ADC_mean_. For assessment of enlarged lymph nodes based on MRI, the agreement between the three readers was only moderate with an overall Fleiss kappa (95% CI) of 0.425 (0.280–0.482). In 8/215 patients, two of the MRI readers recorded MRI-positive LN, whereas the last reader did not. Two of these eight patients were finally staged with LNM.

There were strong-to-moderate positive correlations between the 18F-FDG PET/CT tumor parameters and V_MRI_ (*r* = 0.56–0.83 (range), *p* < 0.001 for all) while only weak negative correlations between ADC_mean_ and the other imaging parameters (*r* = − 0.38–0.43 (range), *p* < 0.001 for all) (Table 2)_._ For the subgroup of 60 patients in whom full 3D tumor segmentation was conducted, the calculated tumor volumes from the 3D method (mean of 14 ml) and the ellipsoid method (mean of 17 ml) yielded an ICC (absolute agreement) of 0.968 (95% CI 0.936–0.982), demonstrating an overall excellent agreement.

**Table 2:** Median (range) values and correlation (Spearman’s rho, *r*) between 18F-FDG PET/CT and MRI tumor markers analyzed in a cohort of 215 endometrial cancer patients

	SUV_max_	SUV_mean_	MTV	V_MRI_	ADC_mean_
Median (range)	14.1 (2.8–39.0)	5.4 (2.3–15.2)	16 (0–744) ml	9 (0–795) ml	779 (383–1665) × 10^−6^ mm^2^/s
	*r* (*n*)	*r* (*n*)	*r* (*n*)	*r* (*n*)	*r* (*n*)
SUV_max_	1 (200)	0.90* (199)	0.72* (200)	0.56* (200)	− 0.40* (192)
SUV_mean_	-	1 (199)	0.70* (199)	0.61* (199)	− 0.38* (191)
MTV	-	-	1 (215)	0.83* (215)	− 0.43* (203)
V_MRI_	-	-	-	1 (215)	− 0.38* (203)
ADC_mean_	-	-	-	-	1 (203)

*SUV*, standardized uptake value; *MTV*, metabolic tumor volume; *V*_*MRI*_, tumor volume from MRI; *ADC*, apparent diffusion coefficient

*Correlation is significant, *p* < 0.001 (2-tailed)

### Association between tumor imaging parameters and clinicopathological patient characteristics

All the F18-FDG PET/CT tumor parameters had significantly higher primary tumor values in patients with DMI, high histological grade (endometrioid grade 3), and advanced stage (FIGO III and IV) (*p* ≤ 0.001 for all; Table 3). SUV_max_ and MTV were also significantly higher in patients with LNM (*p* = 0.04 and *p* < 0.001, respectively), while MTV was the only F18-FDG PET/CT imaging parameter that was significantly associated with CI (*p* = 0.003) (Table 3).

**Table 3:** 18F-FDG PET/CT and MRI tumor markers in relation to surgical and histological tumor characteristics in 215 endometrial cancer patients

	SUV_max_ (*n* = 200^‡^)			SUV_mean_ (*n* = 199^§^)			MTV (ml) (*n* = 215)			V_MRI_ (ml) (*n* = 215)			ADC_mean_ (10^−6^ mm^2^/s) (*n* = 203**)		
Variable	*n*	Median (95% CI)	p*	*n*	Median (95% CI)	p*	*n*	Median (95% CI)	p*	*n*	Median (95% CI)	p*	*n*	Median (95% CI)	p*
Myometrial invasion^†^			*< 0.001*			*< 0.001*			*< 0.001*			*< 0.001*			*< 0.001*
< 50%	125	12.3 (10.6–14.2)		124	4.8 (4.4–5.4)		140	11 (9–14)		140	5 (3–7)		131	808 (783–856)	
≥ 50%	72	16.6 (14.4–18.1)		72	6.4 (5.5–6.8)		72	26 (22–37)		72	16 (11–20)		69	715 (672–742)	
Cervical stroma invasion^†^			0.15			0.16			*0.003*			*0.03*			0.89
No	165	13.2 (12.1–15.0)		164	5.2 (4.9–5.6)		180	15 (13–17)		180	8 (6–9)		170	778 (737–797)	
Yes	31	15.9 (12.3–19.0)		31	6.0 (4.9–6.6)		31	27 (16–43)		31	11 (8–19)		29	787 (736–820)	
Lymph node metastases^†^			*0.04*			0.12			*< 0.001*			*0.001*			0.69
No	115	14.4 (13.0–16.1)		114	5.6 (5.2–6.3)		122	16 (13–18)		122	9 (7–10)		117	781 (735–804)	
Yes	16	17.9 (12.1–25.0)		16	6.6 (4.8–8.8)		16	43 (28–101)		16	27 (11–61)		16	762 (655–851)	
Histological type^†^			0.93			0.91			0.63			0.40			0.95
Endometrioid	161	13.8 (12.5–15.9)		160	5.3 (4.9–5.6)		172	16 (13–19)		172	9 (6–10)		162	775 (737–793)	
Non-endometrioid	35	14.2 (11.8–17.8)		35	5.5 (4.6–6.4)		39	16 (10–37)		39	10 (5–16)		37	797 (738–823)	
Histological grade (endometrioid)^†^			*< 0.001*			*< 0.001*			*< 0.001*			*< 0.001*			*< 0.001*
Grade 1 + 2	131	12.7 (11.0–14.4)		130	5.0 (4.7–5.4)		140	14 (11–17)		140	8 (4–9)		130	784 (755–820)	
Grade 3	26	20.3 (17.0–23.6)		26	7.5 (6.2–8.0)		27	35 (17–94)		27	23 (14–49)		27	676 (598–735)	
FIGO stage^†^			*< 0.001*			*0.001*			*< 0.001*			*< 0.001*			*0.03*
Stage I + II	175	13.2 (12.3–14.9)		174	5.2 (4.9–5.5)		189	15 (13–17)		189	8 (5–9)		177	785 (756–808)	
Stage III + IV	25	18.0 (14.1–21.6)		25	6.9 (5.6–8.0)		26	53 (29–70)		26	27 (14–51)		26	730 (655–785)	

*SUV*, standardized uptake value; *MTV*, metabolic tumor volume; *V*_*MRI*_, tumor volume from MRI; *ADC*, apparent diffusion coefficient; *CI*, confidence interval; *FIGO*, International Federation of Gynecology and Obstetrics

*Mann-Whitney *U* test, two-tailed. Significant *p* values are given in italics

^†^Findings from surgical and pathological staging. Three of the patients did not undergo surgical treatment and one patient underwent tumor debulking. For these patients, final FIGO stages are based on uterine biopsy

^‡^Of the 215 patients in the cohort, 200 patients had increased 18F-FDG uptake in the uterus

^§^One of the 200 patients with increased 18F-FDG uptake in the uterus had SUV_max_ < 2.5, and hence, no SUV_mean_ value could be calculated

**For 12 patients, diffusion-weighted MRI with generation of ADC maps were not possible to perform

V_MRI_ was significantly higher in patients with DMI (*p* < 0.001), CI (*p* = 0.03), LNM (*p* = 0.001), high histological grade (*p* < 0.001), and advanced FIGO stage (*p* < 0.001). Low tumor ADC_mean_ was significantly associated with DMI (*p* < 0.001), high histological grade (*p* < 0.001), and advanced FIGO stage (*p* = 0.03) (Table 3).

### Prediction of lymph node metastases by preoperative imaging parameters

**Fig. 2:**
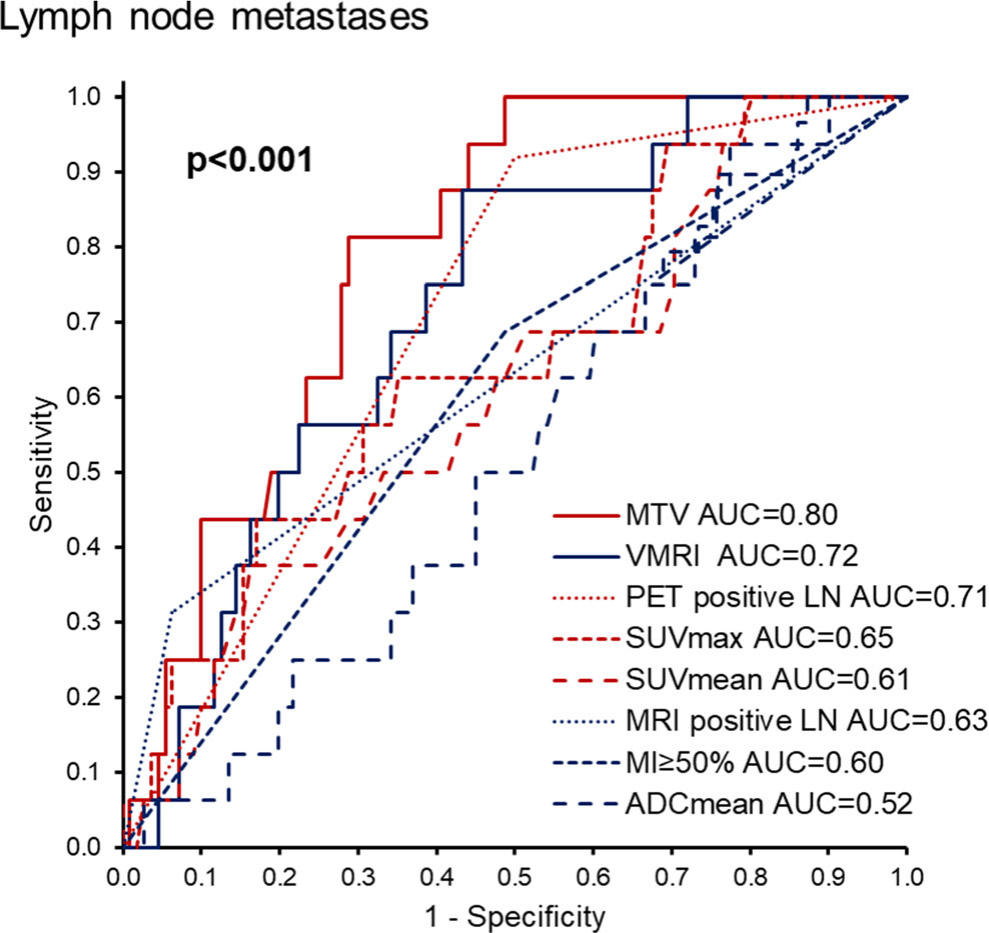
Receiver operating characteristic (ROC) curves for prediction of lymph node metastases for 18F-FDG PET/CT- and MRI-based tumor markers. ADC_mean_, mean tumor apparent diffusion coefficient; MI ≥ 50%, MRI-assessed myometrial invasion ≥ 50%; MRI-positive lymph nodes (LN), enlarged LN based on conventional MRI reading (short-axis diameter ≥ 10 mm); MTV, metabolic tumor volume; SUV_max_, maximum tumor standardized uptake value; SUV_mean_, mean tumor standardized uptake value; PET-positive LN, LN with SUV_max_ ≥ 2.5; V_MRI_, MRI-based tumor volume. *p* value refers to test of equal AUC values across tumor imaging parameters

MTV yielded the highest area under the ROC curve (AUC) for prediction of LNM (AUC = 0.80; Fig. 2). The AUC for predicting LNM was significantly higher for MTV compared with that for V_MRI_, yielding the second highest AUCs (*p* = 0.03). Based on the ROC curves (Fig. 2), the optimal cutoff values for MTV and V_MRI_ were 27 ml and 10 ml, respectively. Using these cutoff values for predicting LNM, MTV > 27 ml yielded significantly higher specificity (*p* < 0.001) and accuracy (*p* < 0.001) while similar sensitivity (*p* = 1.00) to V_MRI_ > 10 ml (Table 4). Furthermore, MTV > 27 ml yielded an odds ratio (OR) of 12.2 (*p* < 0.001), whereas V_MRI_ > 10 ml yielded an OR of 9.7 (*p* = 0.003) for prediction of LNM (Table 4).

**Table 4:** Sensitivity, specificity, PPV, NPV, accuracy, and OR for prediction of pelvic lymph node metastases in *n* = 138 endometrial cancer patients subjected to lymphadenectomy, by MTV larger than 27 ml, V_MRI_ larger than 10 ml, and detected lymph nodes (LN) from conventional 18F FDG PET/CT reading and MRI reading

	MTV > 27 ml*	V_MRI_ > 10 ml*		PET-positive LN	MRI-positive LN	
Sensitivity (%, no. of patients)	81 (13/16)	88 (14/16)	*p* = 1.00^§^	50 (8/16)	31 (5/16)	*p* = 0.25^§^
Specificity (%, no. of patients)	74 (90/122)	58 (71/122)	*p < 0.001*^§^	93(113/122)	94 (115/122)	*p* = 0.77^§^
PPV (%)	29	22		47	42	
NPV (%)	97	97		93	91	
Accuracy (%)	75	62	*p < 0.001*^§^	88	87	*p* = 1.00^§^
Univariate OR (95% CI)	12.2 (3.3–45.6)	9.7 (2.1–44.8)		12.6 (3.8–41.4)	7.5 (2.0–27.5)	
	*p < 0.001*^†^	*p = 0.003*^†^		*p < 0.001*^†^	*p = 0.003*^†^	
Multivariate OR^‡^ (95% CI)	4.5 (0.7–27.6)	2.4 (0.3–20.3)		7.8 (1.7–35.1)	1.5 (0.2–9.1)	
	*p* = 0.10^†^	*p* = 0.41^†^		*p = 0.008*^†^	*p* = 0.67^†^	

Significant *p* values are given in italics

*PPV*, positive predictive value; *NPV*, negative predictive value; *OR*, odds ratio; *MTV*, metabolic tumor volume; *V*_*MRI*_, tumor volume measured from MRI; *CI*, confidence interval

*Optimal cutoff value for MTV and V_MRI_ based on the receiver operating characteristic analysis (Youden’s index) for prediction of lymph node metastases

^†^Binary logistic regression analyses

^‡^Adjusted for risk status based on preoperative endometrial biopsy/curettage indicating endometrioid grade 3 or non-endometrioid histology, in addition to the listed imaging variables

^§^McNemar’s test (*p*, two-tailed) for comparison of sensitivity, specificity, and accuracy between MTV > 27 ml versus V_MRI_ > 10 ml and PET-positive LN versus MRI-positive LN, respectively

The sensitivity for PET-positive LN was higher than for MRI-positive LN (50% vs 31%, respectively), although not statistically significant (*p* = 0.25) (Table 4). The specificity and accuracy were similar for PET- and MRI-positive LN, but PET-positive LN yielded an OR of 12.6 (*p* < 0.001) while MRI-positive LN yielded an OR of 7.5 (*p* = 0.003) (Table 4).

In multivariate analyses including imaging variables as well as preoperative high-risk status (endometrial biopsy/curettage indicating endometrioid grade 3/non-endometrioid histology vs endometrioid grades 1–2), only PET-positive LN remained an independent significant predictor of LNM (OR = 7.8, *p* = 0.008), while MTV > 27 ml tended to the same (OR = 4.5, *p* = 0.10) (Table 4).

Stratifying patients according to MTV >/≤ 27 ml resulted in a false positive rate of 26% (32/122) and a false negative rate of 19% (3/16) in predicting LNM. Corresponding figures when based on PET-positive LN yielded a smaller false positive rate of 7% (9/122), however, with a much higher false negative rate of 50% (8/16). Among patients surgically staged with LNM, 69% (11/16) were assessed as LN negative on PET and/or MRI. Applying the volume cutoff values to this patient subgroup demonstrated MTV > 27 ml in 73% (8/11) and V_MRI_ > 10 ml in 82% (9/11).

Combining lymph node visual assessment on PET and MTV >/≤ 27 ml in a unified prediction model yielded similar diagnostic performance metrics compared with that of MTV > 27 ml alone (Suppl. Fig. 1).

### Prediction of progression-free survival by preoperative imaging parameters

In total, 30 out of the 215 (14%) patients experienced progression. Among these, eleven patients received adjuvant chemotherapy and three patients (with inoperable disease) had primary chemotherapy. Hormonal treatment was given to two of the 30 patients, one as primary treatment (due to fertility wishes) and one as adjuvant treatment. Patients with higher MTV and with PET-positive LN and MRI-positive LN had significantly reduced PFS with univariate hazard ratios (HRs) of 1.003 (*p* = 0.017), 4.0 (*p* = 0.001), and 5.6 (*p* < 0.001), respectively. High V_MRI_ was not significantly associated with reduced survival (Table 5). When adjusting for preoperative high-risk status and patient age, PET- and MRI-positive LN remained significantly associated with reduced survival (HR = 3.3, *p* = 0.004; and HR = 4.3, *p* = 0.001, respectively), while MTV was not (Table 5).

**Table 5:** Cox regression analyses of preoperative 18F-FDG PET/CT and MRI markers for prediction of progression-free survival in 215 patients with endometrial cancer

Imaging variables	Univariate HR (95% CI)	*p*	Adjusted^†^ HR (95% CI)	*p*
MTV	1.003 (1.001–1.006)	*0.017*	1.002 (0.999–1.005)	0.13
MTV > 27 ml	3.6 (1.8–7.4)	*< 0.001*	2.7 (1.3–5.7)	*0.010*
PET-positive LN	4.0 (1.8–9.1)	*0.001*	3.3 (1.5–7.5)	*0.004*
V_MRI_	1.002 (0.999–1.005)	*0.18*	-	-
V_MRI_ > 10 ml	4.1 (1.8–9.2)	*0.001*	3.2 (1.4–7.3)	*0.006*
MRI-positive LN	5.6 (2.5–12.7)	*< 0.001*	4.3 (1.9–10.0)	*0.001*

Significant *p* values are given in italics

*HR*, hazard ratio; *CI*, confidence interval; *MTV*, metabolic tumor volume; *V*_*MRI*_, tumor volume from MRI

^†^Adjusted for risk status based on preoperative endometrial biopsy/curettage indicating endometrioid grade 3 or non-endometrioid histology and patient age at primary treatment

**Fig. 3:**
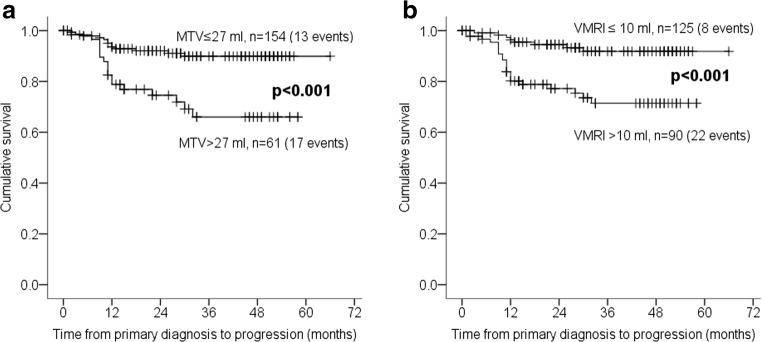
Kaplan-Meier survival curves depicting progression-free survival according to **a** 18F-FDG PET/CT-based metabolic tumor volume (MTV) ≤ 27 versus > 27 ml and **b** MRI-based tumor volume (V_MRI_) ≤ 10 versus > 10 ml. *P* values refer to the log-rank test. For each category: number of cases (number of cases with progression)

When stratifying according to the proposed volume cutoff values for prediction of LNM, both MTV > 27 ml and V_MRI_ > 10 ml were significantly associated with reduced progression-free survival (Fig. 3), both in the univariate analyses (HR = 3.6, *p* < 0.001; and HR = 4.1, *p* = 0.001, respectively) and in multivariate analyses (HR = 2.7, *p* = 0.010; and HR = 3.2, *p* = 0.006, respectively) (Table 5).

In patients surgicopathologically staged as FIGO I–II (*n* = 189), large MTV, V_MRI_ > 10 ml, and MRI-positive LN tended to predict poor PFS with HRs of 1.009 (*p* = 0.07), 2.2 (*p* = 0.09), and 3.9 (*p* = 0.07), respectively (Suppl. Table 3). For patients with FIGO stages III–IV (*n* = 26), none of the imaging markers significantly predicted survival (Suppl. Table 3). Among patients primary staged as FIGO I–II, 10% (19/189) eventually developed progression; in these nineteen patients, 37% (7/19) had MTV > 27 ml and 58% (11/19) had V_MRI_ > 10 ml.

## Discussion

Preoperative identification of LNM and high-risk disease is critical for better tailoring of surgical procedure and adjuvant therapy in endometrial cancer patients. In this large population-based study, we demonstrate that imaging markers from 18F-FDG PET/CT outperform MRI markers for the prediction of LNM. To our knowledge, this is the first study comparing metabolic tumor volume and LNM detected on 18F-FDG PET/CT to anatomical tumor volume and LNM detected on MRI, in a large cohort of endometrial cancer patients.

This study demonstrates that conventional PET/CT reading has high specificity (93%), but limitations in sensitivity (50%) for prediction of LNM. The sensitivity in this study is in the lower range of that reported in previous PET/CT studies on endometrial cancer [19–22, 24]. However, several of these previous studies comprise patient cohorts with higher percentage of patients having advanced stage and with higher prevalence of LNM compared with the present study, which may partly explain the differences in diagnostic performance metrics achieved for the different cohorts. Interestingly, when employing MTV > 27 ml as a cutoff value, higher sensitivity (81%), although at the cost of lower specificity (74%), was achieved. While conventional PET/CT reading has well-known limitations in diagnosing micrometastases, high MTV may pose an increased risk of micrometastases which is likely to explain the higher sensitivity observed for MTV > 27 ml compared with the conventional PET/CT reading for prediction of LNM.

In a recent large study comprising of 287 endometrial cancer patients preoperatively staged by both PET/CT and MRI, LNM detection based on PET/CT yielded better sensitivity than MRI (70% vs 34%) [24]. The same tendency was observed in the present study, with sensitivity of 50% versus 31% for PET- and MRI-positive LN, respectively (Table 4). However, we also found that MTV > 27 ml yield significantly higher specificity, accuracy, and odds ratios for prediction of LNM compared with V_MRI_ > 10 ml, suggesting that MTV measurement may represent a valuable adjunct to the conventional PET/CT reading in endometrial cancer.

For prediction of survival, we found that high MTV, PET-positive LN, and MRI-positive LN significantly predicted reduced progression-free survival whereas high V_MRI_ did not. Interestingly, when stratifying MTV and V_MRI_ according to the proposed cutoff values, both V_MRI_ > 10 ml and MTV > 27 ml were significantly associated with reduced progression-free survival. This is in line with previous endometrial cancer studies reporting that large tumor size measured by MRI [25] and large MTV at 18F-FDG PET/CT [26] both are linked to reduced survival. To our knowledge, this is, however, the first study to compare V_MRI_ and MTV in a large patient cohort and to show that the negative prognostic impact of large MTV may be even higher than that of large V_MRI_ in endometrial cancer.

Several risk models for prediction of LNM have been proposed [7–12], and in a study from 2015, Koskas et al [13] evaluated ten models in an independent patient cohort and found that the best model (based on CA125 and MRI findings [12]) yielded an AUC of 0.76 and a false negative rate of 4%. The present study shows that a risk model based on MTV cutoff value of 27 ml alone will yield a higher AUC of 0.80 for prediction of LNM, however with a higher false negative rate of 19%. Whether a combination of MTV and other preoperative clinical/biochemical/molecular markers may yield even better prediction of LNM remains to be explored in future studies and needs to be validated in independent patient cohorts.

This study has some limitations. First, the imaging protocols at our institution have been revised during the study period. MRI was performed on two different scanners (1.5 and 3 T), and the CT protocol used for attenuation correction of the PET images was changed. The 3-T MRI protocol was, however, intentionally set up to be very similar to the 1.5-T protocol (Suppl. Table 1). Also, the impact on PET attenuation correction due to differences in CT protocols is assumed to be minimal. This assumption is supported by the FDG PET/CT procedure guidelines for tumor imaging [27] which state that contrast agents only minimally affect the SUV quantification. Also, when excluding the 11 patients (with attenuation correction based on diagnostic CT) in the analyses in the present study, all significant findings were reproduced, supporting that the change in attenuation correction protocol has not substantially biased our results. Secondly, the MRI tumor volumes were estimated assuming the tumor shape of an ellipsoid, which might not be the case for all tumors. However, given an ICC of 0.968 between 3D MRI tumor segmentation method and the ellipsoid method in the subgroup of 60 patients, it seems highly unlikely that the employed method for estimation of MRI tumor volumes has substantially biased our results.

In conclusion, this study found that imaging markers from 18F-FDG PET/CT outperform MRI markers for the prediction of LNM in endometrial cancer patients. MTV > 27 ml yielded a high diagnostic performance for predicting LNM and aggressive disease and represents a promising supplement to conventional PET/CT reading in endometrial cancer. However, the promising role of PET/CT-derived biomarkers needs to be confirmed in independent cohorts and evaluated in combination with other preoperative biomarkers and novel emerging techniques such as SLND, in order to define the added value of PET/CT for better prediction of LNM and aggressive disease in endometrial cancer.

## Electronic supplementary material

ESM 1 (DOCX 235 kb)

## Abbreviations

18F-FDG: Fluorine-18 fluorodeoxyglucose
ADC: Apparent diffusion coefficient
CE: Contrast-enhanced
CI: Cervical stroma invasion
DMI: Deep myometrial invasion
FIGO: The International Federation of Gynecology and Obstetrics system
LNM: Lymph node metastases
MRI: Magnetic resonance imaging
MTV: Metabolic tumor volume
PET/CT: Positron emission tomography/computed tomography
SLND: Sentinel lymph node dissection
SUV: Standardized uptake value
V_MRI_: Tumor volume measured on MRI

## References

[1] Bray F, Ferlay J, Soerjomataram I, Siegel RL, Torre LA, Jemal A (2018). Global cancer statistics 2018: GLOBOCAN estimates of incidence and mortality worldwide for 36 cancers in 185 countries. CA Cancer J Clin.

[2] Colombo N, Creutzberg C, Amant F (2015). ESMO-ESGOESTRO Consensus Conference on Endometrial Cancer: diagnosis, treatment and follow-up. Radiother Oncol.

[3] Creasman W (2009). Revised FIGO staging for carcinoma of the endometrium. Int J Gynaecol Obstet.

[4] Benedetti Panici P, Basile S, Maneschi F (2008). Systematic pelvic lymphadenectomy vs. no lymphadenectomy in early-stage endometrial carcinoma: randomized clinical trial. J Natl Cancer Inst.

[5] Kitchener H, Swart AM, Qian Q, Amos C, Parmar MK, ASTEC study group (2009). Efficacy of systematic pelvic lymphadenectomy in endometrial cancer (MRC ASTEC trial): a randomised study. Lancet.

[6] Naumann RW (2012). The role of lymphadenectomy in endometrial cancer: was the ASTEC trial doomed by design and are we destined to repeat that mistake?. Gynecol Oncol.

[7] Creasman WT, Morrow CP, Bundy BN (1987). Surgical pathologic spread patterns of endometrial cancer. A Gynecologic Oncology Group Study. Cancer.

[8] Bendifallah S, Genin AS, Naoura I (2012). A nomogram for predicting lymph node metastasis of presumed stage I and II endometrial cancer. Am J Obstet Gynecol.

[9] AlHilli MM, Podratz KC, Dowdy SC (2013). Risk-scoring system for the individualized prediction of lymphatic dissemination in patients with endometrioid endometrial cancer. Gynecol Oncol.

[10] Todo Y, Sakuragi N, Nishida R (2003). Combined use of magnetic resonance imaging, CA 125 assay, histologic type, and histologic grade in the prediction of lymph node metastasis in endometrial carcinoma. Am J Obstet Gynecol.

[11] Lee JY, Jung DC, Park SH (2010). Preoperative prediction model of lymph node metastasis in endometrial cancer. Int J Gynecol Cancer.

[12] Kang S, Kang WD, Chung HH (2012). Preoperative identification of a low-risk group for lymph nodemetastasis in endometrial cancer: a Korean gynecologic oncology group study. J Clin Oncol.

[13] KoskasM FM, Vanderstraeten A (2016). Evaluation of models to predict lymph node metastasis in endometrial cancer: a multicentre study. Eur J Cancer.

[14] Korkmaz V, Meydanli MM, Yalcin I (2017). Comparison of three different risk-stratification models for predicting lymph node involvement in endometrioid endometrial cancer clinically confined to the uterus. J Gynecol Oncol.

[15] Boyraz G, Atalay FO, Salman MC (2018). Comparison of Mayo and Milwaukee risk stratification models for predicting lymph node metastasis in endometrial Cancer. Int J Gynecol Cancer.

[16] Brugger S, Hamann M, Mosner M (2018). Endometrial cancer how many patients could benefit from sentinel lymph node dissection?. World J Surg Oncol.

[17] Geppert B, Lönnerfors C, Bollino M, Persson J (2018). Sentinel lymph node biopsy in endometrial cancer-feasibility, safety and lymphatic complications. Gynecol Oncol.

[18] Haldorsen IS, Salvesen HB (2016). What is the best preoperative imaging for endometrial cancer?. Curr Oncol Rep.

[19] Kakhki VRD, Shahriari S, Treglia G (2013). Diagnostic performance of fluorine 18 fluorodeoxyglucose positron emission tomography imaging for detection of primary lesion and staging of endometrial cancer patients: systematic review and meta-analysis of the literature. Int J Gynecol Cancer.

[20] Husby JA, Reitan BC, Biermann M (2015). Metabolic tumor volume on 18F-FDG PET/CT improves preoperative identification of high-risk endometrial carcinoma patients. J Nucl Med.

[21] Bollineni VR, Ytre-Hauge S, Bollineni-Balabay O (2016). High diagnostic value of 18F-FDG PET/CT in endometrial cancer:systematic review and meta-analysis of the literature. J Nucl Med.

[22] Atri M, Zhang Z, Dehdashti F (2017). Utility of PET/CT to evaluate retroperitoneal lymph node metastasis in high-risk endometrial cancer: results of ACRIN 6671/GOG 0233 trial. Radiology.

[23] Gee MS, Atri M, Bandos AI (2018). Identification of distant metastatic disease in uterine cervical and endometrial cancers with FDG PET/CT: analysis from the ACRIN 6671/GOG 0233 multicenter trial. Radiology.

[24] Kim HJ, Cho A, Yun M (2016). Comparison of FDG PET/CT and MRI in lymph node staging of endometrial cancer. Ann Nucl Med.

[25] Ytre-Hauge S, Husby JA, Magnussen IJ (2015). Preoperative tumor size at MRI predicts deep myometrial invasion, lymph node metastases, and patient outcome in endometrial carcinomas. Int J Gynecol Cancer.

[26] Shim S-H, Kim D-Y, Lee D-Y (2014). Metabolic tumour volume and total lesion glycolysis, measured using preoperative 18F–FDG PET/CT, predict the recurrence of endometrial cancer. BJOG.

[27] Boellaard R, Delgado-Bolton R, WJG O (2015). FDG PET/CT: EANM procedure guidelines for tumour imaging: version 2.0. Eur J Nucl Med Mol Imaging.

