# Supplementary material for: Preoperative 18F-FDG PET/CT tumor markers outperform MRI-based markers for the prediction of lymph node metastases in primary endometrial cancer
Source: Eur Radiol. 2020 Feb 7;30(5):2443–53. doi: 10.1007/s00330-019-06622-w (PMC7160067; doi:10.1007/s00330-019-06622-w)
Supplement: Supplementary file 1 — (DOCX 235 kb) [file 330_2019_6622_MOESM1_ESM.docx]

| **SUPPL. TABLE 1** MRI scanning protocols | | | | | | | | |
| --- | --- | --- | --- | --- | --- | --- | --- | --- |
| **MR scanner** | **Sequence** | **Plane** | **TR/TE_1_/TE_2_ (ms)** | **FA (deg)** | **Slice/**  **Incr (mm)** | **Acquisition matrix** | **FOV (mm^3^)** | **Pixel size (mm^2^)** |
| 1.5T  Siemens Avanto | T1 VIBE +C | PA | 7.23/2.55 | 20 | 2.0/2.0 | 192x154 | 250x250 | 1.6x1.3 |
|  | T2 TSE | PA | 6310/95 | 150 | 3.0/3.3 | 256x205 | 180x180 | 0.9x0.7 |
|  | T2 TSE | SAG | 4920/95 | 150 | 3.0/3.0 | 256x205 | 180x180 | 0.9x0.7 |
|  | DWI | PA | 3100/79 | 90 | 5.0/6.0 | 128x128 | 300x300 | 2.3x2.3 |
| 3T  Siemens Skyra | T1 VIBE DIXON +C | PA | 5.86/2.46/3.69 | 9 | 1.2/1.2 | 139x256 | 250x250 | 1.0x1.0 |
|  | T2 TSE | PA | 4330/94 | 150 | 3.0/3.3 | 326x384 | 200x200 | 0.5x0.5 |
|  | T2 TSE | SAG | 7360/101 | 160 | 3.0/3.3 | 310x320 | 200x200 | 0.6x0.6 |
|  | DWI RESOLVE | PA | 6010/74/126 | 180 | 3.0/3.3 | 144x144 | 200x200 | 1.4x1.4 |
| C, contrast enhanced. Deg, degrees. DWI, diffusion weighted imaging. Incr, increment between slice positions. FA, flip angle. FOV, field of view. PA, paraxial slice orientation. RESOLVE, Readout Segmentation Of Long Variable Echo trains. SAG, sagittal slice orientation. TE, time echo. TR, repetition time. TSE, turbo spin echo. VIBE, volumetric interpolated breath-hold examination. | | | | | | | | |

| **SUPPL. TABLE 2** Patient characteristics, clinicopathological findings, imaging findings, treatment and outcome (progression) in 215 endometrial cancer patients with and without lymphadenectomy | | | | | | |
| --- | --- | --- | --- | --- | --- | --- |
|  | | | **Patients with lymphadenectomy (n=138)** | **Patients without lymphadenectomy (n=77)** | | **p^††^** |
| Age, mean (range) | | | 68 (41-88) | 67 (30-90) | | 0.57 |
| BMI, mean (range) | | | 27 (16-50) | 31 (20-53) | | **0.001** |
| Postmenopausal, n (%) | | | 136 (99%) | 68 (88%) | | **0.001** |
| PET positive LN^‡^ | | |  |  | | **0.04** |
| No | | | 121 (88%) | 74 (96%) | |  |
| Yes | | | 17 (12%) | 3 (4%) | |  |
| MTV>27 ml^‡^ | | |  |  | | 0.07 |
| No | | | 93 (67%) | 61 (79%) | |  |
| Yes | | | 45 (33%) | 16 (21%) | |  |
| MRI positive LN^§^ | | |  |  | | 0.60 |
| No | | | 126 (91%) | 72 (94%) | |  |
| Yes | | | 12 (9%) | 5 (6%) | |  |
| V_MRI_>10 ml^§^ | | |  |  | | **0.04** |
| No | | | 73 (53%) | 52 (68%) | |  |
| Yes | | | 65 (47%) | 25 (32%) | |  |
| Risk status* from preoperative biopsy/curettage | | |  |  | | **<0.001** |
| Low-risk | | | 73 (53%) | 71 (92%) | |  |
| High-risk | | | 65 (47%) | 6 (8%) | |  |
| FIGO stage^†^, n (%) | | |  |  | | 0.06 |
| Stage I+II | | | 117 (85%) | 72 (94%) | |  |
| Stage III+IV | | | 21 (15%) | 5 (6%) | |  |
| Histologic subtype^†^, n (%) | | |  |  | | **<0.001** |
| Endometrioid | | | 97 (70%) | 75 (98%) | |  |
| Non-endometrioid | | | 38 (28%) | 1 (1%) | |  |
| Undifferentiated/other | | | 3 (2%) | 1 (1%) | |  |
| Histologic grade^†^ (endometrioid only), n (%) | | |  |  | | **<0.001** |
| Grade 1 | | | 70 (72%) | 70 (93%) | |  |
| Grade 2 | | | 24 (25%) | 3 (4%) | |  |
| Grade 3 | | | 3 (3%) | 2 (3%) | |  |
| Myometrial invasion^†^, n (%) | | |  |  | | **0.002** |
| < 50% | | | 81 (59%) | 59 (77%) | |  |
| ≥ 50% | | | 57 (41%) | 15 (19%) | |  |
| Missing | | | 0 (0%) | 3 (4%) | |  |
| Cervical stroma invasion^†^, n (%) | | |  |  | | **0.02** |
| No | | | 112 (81%) | 68 (88%) | |  |
| Yes | | | 26 (19%) | 5 (7%) | |  |
| Uterus not removed | | | 0 (0%) | 4 (5%) | |  |
| Primary surgical treatment | | |  |  | | **0.03** |
| Hysterectomy | | | 138 | 73 | |  |
| Tumor debulking | | | 0 | 1 | |  |
| No | | | 0 | 3 | |  |
| Adjuvant treatment | | |  |  | | **<0.001** |
| Chemotherapy | | | 66 (48%) | 6 (8%) | |  |
| Hormonal treatment | | | 1 (1%) | 1 (1%) | |  |
| External radiation | | | 2 (1%) | 0 (0%) | |  |
| Internal radiation | | | 0 (0%) | 2 (3%) | |  |
| No | | | 69 (50%) | 68 (88%) | |  |
| Progression** | | |  |  | | **0.05** |
| No | | | 114 (83% ) | 71 (92%) | |  |
| Yes | | | 24 (17%) | 6 (8%) | |  |
| BMI, body mass index. FIGO, International Federation of Gynecology and Obstetrics.  *Low-risk: Endometrioid subtype grade 1 and 2. High-risk: Endometrioid subtype grade 3 and non-endometrioid subtype.  ^†^Findings from surgical and pathological staging.  ^‡^Findings from preoperative whole-body 18F-FDG PET/CT  ^§^Findings from preoperative pelvic MRI  **Progression defined as local recurrence/progression in the pelvis or new metastases in the abdomen or at distant sites.  ^††^Pearson Chi-Square test for categorical variables. Mann-Whitney U-test for continuous variables. | | | | | | |
| **SUPPL. TABLE 3** Cox regression analyses of preoperative 18F-FDG PET/CT and MRI markers for prediction of progression-free survival in FIGO stage I-II and FIGO stage III-IV endometrial cancer patients | | | | |  |  |
| **FIGO** | **Imaging variables** | **Univariate HR (95% CI)** | | **p** |  |  |
| I-II (n=189) | MTV | 1.009 (0.999-1.018) | | 0.07 |  |  |
|  | MTV >27 ml | 1.8 (0.7-4.7) | | 0.24 |  |  |
|  | PET positive LN | 1.1 (0.2-8.5) | | 0.90 |  |  |
|  | V_MRI_ | 1.01 (1.00-1.03) | | 0.21 |  |  |
|  | V_MRI_ >10 ml | 2.2 (0.9-5.6) | | 0.09 |  |  |
|  | MRI positive LN | 3.9 (0.9-17.1) | | 0.07 |  |  |
| III-IV (n=26) | MTV | 1.000 (0.995-1.004) | | 0.96 |  |  |
|  | MTV >27 ml | 3.4 (0.4-27.0) | | 0.25 |  |  |
|  | PET positive LN | 2.5 (0.7-8.7) | | 0.15 |  |  |
|  | V_MRI_ | 0.998 (0.991-1.005) | | 0.55 |  |  |
|  | V_MRI_ >10 ml | coeff. did not converge | | - |  |  |
|  | MRI positive LN | 2.3 (0.6-8.2) | | 0.21 |  |  |
| FIGO, International Federation of Gynecology and Obstetrics. HR, hazard ratio. CI, confidence interval. MTV, metabolic tumor volume. V_MRI_, tumor volume from MRI.  Significant p values are given in boldface. | | | | |  |  |

| **SUPPL. TABLE 4** Cox regression analyses of preoperative 18F-FDG PET/CT and MRI markers for prediction of progression-free survival in 215 patients with endometrial cancer | | | | |
| --- | --- | --- | --- | --- |
| **Imaging variables** | **Univariate HR (95% CI)** | **p** | **Adjusted**^†^ **HR (95% CI)** | **p** |
| MTV | 1.003 (1.001-1.006) | **0.017** | 1.002 (0.999-1.005) | 0.28 |
| MTV >27 ml | 3.6 (1.8-7.4) | **<0.001** | 2.01 (0.93-4.33) | 0.07 |
| PET positive LN | 4.0 (1.8-9.1) | **0.001** | 2.4 (1.1-5.6) | **0.04** |
| V_MRI_ | 1.002 (0.999-1.005) | 0.18 | - | - |
| V_MRI_ >10 ml | 4.1 (1.8-9.2) | **0.001** | 2.4 (1.0-5.6) | **0.05** |
| MRI positive LN | 5.6 (2.5-12.7) | **<0.001** | 3.4 (1.5-8.0) | **0.004** |
| HR, hazard ratio. CI, confidence interval. MTV, metabolic tumor volume. V_MRI_, tumor volume from MRI.  Significant p values are given in boldface.  ^†^Adjusted for risk status based on preoperative endometrial biopsy/curettage indicating endometrioid grade 3 or non-endometrioid histology, patient age at primary treatment and adjuvant treatment. | | | | |

**
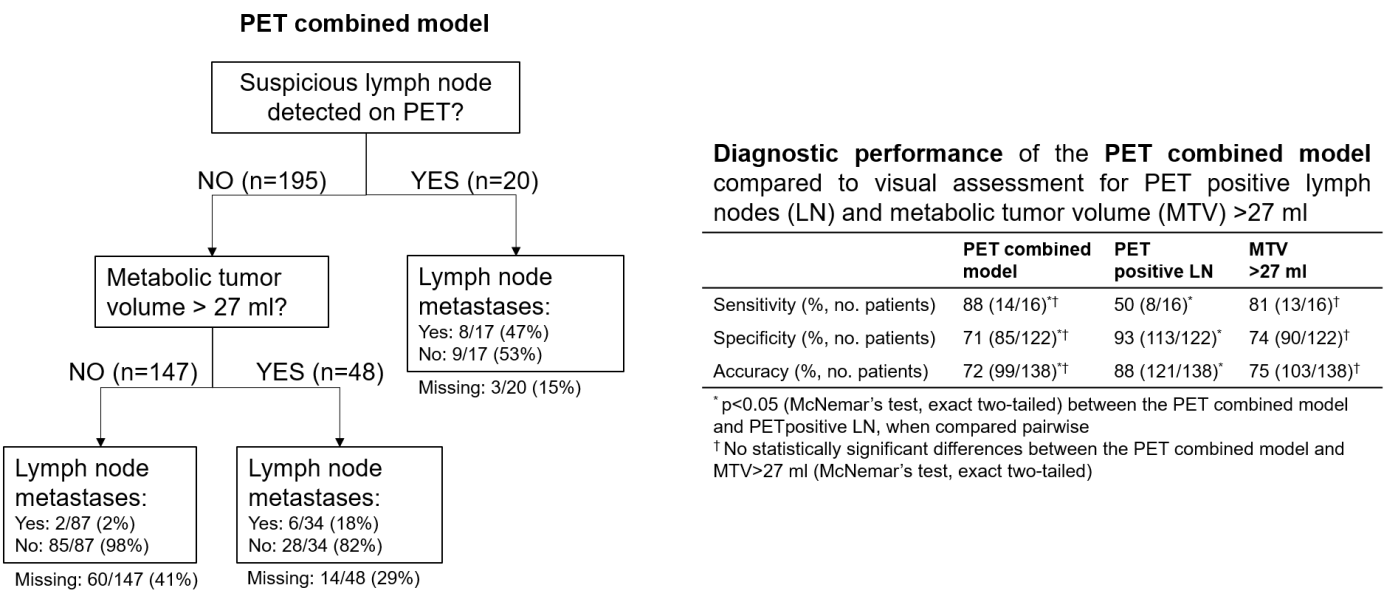
**

**SUPPL. FIG 1** Model for combining lymph node visual assessment and metabolic tumor volume (MTV)>27 ml in 18F-FDG PET-CT of 215 endometrial cancer patients. Final lymph node metastases status (yes/no) is given for the 138 patients who underwent lymphadenectomy and the status “missing” is given for patients who did not undergo lymphadenectomy (n=77) (flowchart to the left). Sensitivity, specificity and accuracy are given for the PET combined model, visual assessment alone (PET positive LN) and MTV>27 ml alone (table to the right). PET combined model yielded similar diagnostic performance metrics compared to that of MTV>27 ml alone.
